# Assessment of *Bacillus* species capacity to protect Nile tilapia from *A. hydrophila* infection and improve growth performance

**DOI:** 10.3389/fcimb.2024.1354736

**Published:** 2024-07-09

**Authors:** Leslye Macias, Víctor Mercado, Jorge Olmos

**Affiliations:** Molecular Microbiology Laboratory, Department of Marine Biotechnology, Centro de Investigación Científica y de Educación Superior de Ensenada, Ensenada, Baja California, Mexico

**Keywords:** *Aeromonas hydrophila*, *Bacillus*, Nile tilapia, probiotics, functional foods

## Abstract

The present study evaluated the capacity of three *Bacillus* species to improve health status and growth performance of Nile Tilapia fed with high levels of soybean meal and challenged with *Aeromonas hydrophila*. *In vitro* experiments showed that β-hemolysin and metalloprotease enzymes were produced *by A. hydrophila* throughout the exponential growth phase. *In vivo* experiments showed that 10^7^ colony-forming units (CFUs)/ml of this pathogen killed 50% of control group fishes in 13 days. To evaluate the influence of *Bacillus* strains on health status and growth performance in Nile Tilapia, 180 fishes (33.44 + 0.05 g) were distributed in 12 tanks of 200 L each, and animals were fed twice per day until satiety. 1) Control group without *Bacillus*, 2) *Bacillus* sp1, 3) *Bacillus* sp2, and 4) *Bacillus* sp3 groups were formulated containing 10^6^ CFU/g. After 40 days of feeding, the fishes were intraperitoneally injected with 1 ml of *A. hydrophila* at 2 × 10^7^ CFU/ml, and mortality was recorded. The results showed that cumulative mortality rate was significantly (p< 0.05) lower in the *Bacillus* sp1 (25%), sp2 (5%), and sp3 (15%) groups, than the control group (50%). Weight gain was also significantly better (p< 0.05) in the *Bacillus* sp1 (36%), sp2 (67%), and sp3 (55%) groups with respect to the control group (30%). In conclusion, functional diet formulated with high levels of soybean meal and supplemented with *Bacillus* sp2 could be an alternative to protect Nile tilapia cultures from *A. hydrophila* infections and improve fish growth performance.

## 1 Introduction

Fish global demand has been growing in the last decades, and to satisfy present requirements, the aquaculture sector has developed several alternatives to improve its production. Intensive systems are capable of increasing fish production, but also increases fish susceptibility to bacterial infections due to stress induced on animals (Bondad-Reantaso et al., 2005; Makled et al., 2019). *Aeromonas hydrophila* is one of the most important pathogens in freshwater fish production. This bacterium is responsible for disease outbreaks and economical losses in aquaculture systems (Řehulka, 2002; Kumar, 2005; Abdel-Latif and Khafaga, 2020). *A. hydrophila* could produce toxins, hemolysins, and proteases, which are responsible for inducing systemic damage and fish death (Beaz-Hidalgo and Figueras, 2013; Tomás, 2012; El-Bahar et al., 2019). There is not enough information about this pathogen behavior; therefore, some questions arise as follows: 1) Which optimum conditions could grow it (growth medium, temperature, pH, aeration rate, etc.)? 2) How do these conditions influence virulence factor (VF) production? 3) When does VF initiate and finish its production? 4) How long (time) does VF reach their maximal concentration? Nowadays, antibiotics and other chemicals are the main compounds used for disease control in aquaculture; however, this strategy has induced pathogen resistance and food safety problems (Angulo et al., 2004; Cabello, 2006). New strategies to replace antibiotics in the aquaculture industry are being investigated to prevent and control disease outbreaks. Functional feeds formulated with probiotics and high levels of vegetable ingredients have been a successful alternative to improve the health status and growth performance of animals (Olmos et al., 2022). The *Bacillus* species are being used as probiotic bacteria in aquafeed formulations due to their growth capacity in different nutrient sources, tolerance for extreme environmental conditions, secretion of high levels of enzymes, production of antimicrobial compounds, and their being considered as Generally recognized as safe (GRAS) by the Food and Drug Administration (FDA) (Olmos and Paniagua Michel, 2014; Soltani et al., 2019; Kuebutornye et al., 2020a). Recently, the *Bacillus* species have been used in Nile Tilapia diets to protect it against *A. hydrophila* infections (Mehisan et al., 2015; Gobi et al., 2018; Naser et al., 2019; Kuebutornye et al., 2020b; Won et al., 2020; Xu et al., 2022). The results show that the addition of *Bacillus* to those diets improved health status and growth performance of the fish. In this work, Nile Tilapia weight gain and survival percentage were improved with respect to previous reports when *Bacillus* sp2 strain was included in feed formulated with high levels of soybean meal and challenged with *A. hydrophila* pathogen strain.

## 2 Materials and methods

### 2.1 *Aeromonas hydrophila* characterization

#### 2.1.1 DNA purification

*A. hydrophila* CAIM675 strain was isolated from mouth lesions of rainbow trout at the Food and Development Research Center (CIAD, Mazatlán, Mexico). Bacterium was grown at 30°C in brain hart infusion medium (BHI) for 12 h at 200 rpm and centrifuged at 12,000 × *g* for 3 min. The pellet was suspended in 567 μl of Buffer TE and incubated with 5 μl of lysozyme and 10 μl of RNAasa. Three microliters of proteinase K and 30 μl of Sodium dodecyl sulfate (SDS) were added, and the sample was incubated for 1 h at 37°C. One hundred microliters of NaCl 5 M and 80 μl of Cetyltrimethylammonium bromide (CTAB)/NaCl were added, and the sample was incubated for 10 min at 65°C. To remove CTAB–protein/polysaccharides, isoamyl alcohol/chloroform solution was added and vortex was applied. Isoamyl alcohol/phenol–chloroform solution was added, vortexed, and centrifuged for 5 min. To DNA precipitation, 360 μl of isopropanol was added, and the sample was then mixed and centrifuged. Ethanol at 70% was used to wash and purify DNA; the sample was centrifuged and dried by Vacufuge for 10 min at 30°C. The pellet was suspended in Buffer TE and stored at −20°C (Sambrook et al., 1989).

#### 2.1.2 Virulence gene identification

PCR reactions were carried out in a 25-μl final volume using 12.5 μl of Taq 2× Master mix, 0.5 μl of each primer, 0.5 μl of DNA, and 11 μl of H_2_O dd. The amplification was performed using 25 cycles in a BioRad iCycler Thermal Cycler with a melting temperature (Tm) of 53°C to all reactions. One cycle consisted of 1 min at 95°C, 1 min at 53°C, and 2 min at 72°C. To identify virulence genes, primers in **Table 1** were designed. PCR amplification was analyzed through electrophoresis using 1.2% agarose gel.

**Table 1:** Primers used for virulence genes detection in *Aeromonas hydrophila* CAIM675.

Gen	Primer	Access no.	Product
*act*	AactF-GCAATCACAGCCAATATGTC AactR-CCACTTGAACTTGTTCTTGG	KC687134.1	353pb
*hylA*	AhylAF-GCAATCACAGCCAATATGTC AhylAR-GAGATGTCAGCCTTGTAGAG	FJ380998.1	520pb
*ahpB*	AahpBF-GAGAACTACATGAAGGGCAG AahpBR-CAGGCTAAATCACATCAACC	AF193422.1	717pb
*ast*	AstF-CAACATATCTGGGTAGCTGG AstR-CTATGAGGTCATCTTCCTGC	AF419157.1	534pb
*ser*	AserF-CAGGCAATGATCTCAACCTC AserR-GGGTCTATGTTGCTGTTCTC	AY841795.1	586pb
*aerA*	AaerF-CCTGGATATTCCAGATGGTG AaerR-CTATCTTCACCGGGATCTTG	M16495.1	617pb

#### 2.1.3 Growth conditions and virulence factors production

*A. hydrophila* was grown at 30°C and 200 rpm for 12 h in Erlenmeyer flasks of 250-ml capacity containing 30 ml of Brain-heart infusion (BHI) medium (MCD LAB, Mexico). New flasks with 27 ml of BHI medium were inoculated with 3 ml of preinoculum mentioned above and grown at the same conditions. Bacterium growth was followed each hour at 600 nm, and samples were collected every 2 h. A supernatant obtained through centrifugation at 12,000 × *g* for 10 min at 4°C was passed through a sterile 0.2-µm filter (Supor^®^ 200 Membrane Disc Filters, 0.2 µm to 13 mm, plain 100/pkg; Pall Corporation), and 1 ml of the sample was stored at −20°C. Hemolytic activity was evaluated applying 40 μl of filtered sample onto blood–agar plates. Proteolytic activity was evaluated applying 40 μl of filtered sample onto skim milk agar plates. All experiments were performed in triplicate, and plates were incubated at 30°C for 24 h. The degradation zone around colonies was measured in mm^2^.

#### 2.1.4 Median lethal dose identification (LD_50_)

LD_50_ in Nile Tilapia was estimated by following the method of Reed and Muench (1938) with slight modifications: *A. hydrophila* was grown in BHI medium at 30°C and 200 rpm for 6 h, optical density was measured at 600 nm, and colony-forming units (CFUs) were obtained in agar plates. Subsequently, 10 fishes weighing 60 ± 0.5 g were intraperitoneally injected with 1 ml of *A. hydrophila* at 2 × 10^7^ CFU/ml. Fishes were monitored, and typical infection symptoms were identified and recorded according to Yardimci and Aydin (2011).

### 2.2 *Bacillus* strain characterization

#### 2.2.1 *Bacillus* strain identification

*Bacillus* strains used in this research were obtained from our laboratory collection at the Ensenada Center for Scientific Research and Higher Education (CICESE, Ensenada, Baja California, Mexico). Three strains named sp1, sp2, and sp3 were selected for their capacity to grow in soy-based media and inhibit *A. hydrophila* development in agar plates. For molecular identification, sp1, sp2, and sp3 strains were grown in LB medium for 12 h to DNA extraction. DNA purification was carried out by standard phenol:chloroform:isoamyl alcohol (25:24:1) procedure, and its quality was evaluated by agarose gel (1.2% w/v) electrophoresis. 16S rDNA gene amplification was performed using *Bacillus*-specific oligonucleotides F (5′-ACAGAGTTTGATCCTGGCTCAG-3′) and R (5′-CCCAGTTTCCAATGACC-3′) previously described (Arellano and Olmos, 2002), and products were sequenced by the Institute of Biotechnology (IBT) of the National Autonomous University of Mexico (UNAM). Strain identity was determined by the NCBI database using 16S rDNA gene and Basic Local Alignment Search Tool (BLAST). Phylogenetic identification analysis was performed using the MEGA X program.

#### 2.2.2 Antimicrobial activity against *A. hydrophila*

Antimicrobial activity was evaluated making co-cultures of *Bacillus* sp1, sp2, and sp3 strains with *A. hydrophila* in BHI agar plates at 30°C for 24 h. Inhibition zone was measured in mm^2^ and recorded.

#### 2.2.3 *Bacillus* strain enzymatic activity

Protease, carbohydrase, and lipase activity was measured according to Ochoa-Solano and Olmos-Soto (2006). Protease production was evaluated using skim milk and soy agar plates. Carbohydrase activity was tested using a medium with starch, corn meal, or wheat flour. Lipase production was evaluated using olive oil at 2.5% (w/v) and Rhodamine B solution at 0.001% (w/v). All plates were incubated at 37°C; bacterial growth and degradation zone around colonies were measured after 24 h.

#### 2.2.4 Antibiotic resistance

Following the CSLI (Clinical and Laboratory Standards Institute) and EFSA (European Food Safety Authorities) guidelines, *Bacillus* strains and *A. hydrophila* were tested for resistance to ampicillin, chloramphenicol, oxytetracycline, erythromycin, streptomycin, clindamycin, cephalexin, and doxycycline. Plates were incubated for 24 h at 37°C for *Bacillus* and 30°C for *A. hydrophila*.

#### 2.2.5 *Bacillus* strain growth

*Bacillus* strains were grown in Luria-Bertani (LB) agar plates at 37°C for 12 h, and Erlenmeyer flasks containing 30 ml of LB broth were inoculated and incubated at 37°C and 250 rpm for 12 h. Three milliliters of preinoculum mentioned above were inoculated in 27 ml of Schaffer medium, and growth was permitted at conditions mentioned above. *Bacillus* strain growth was measured each hour at 600 nm, and samples were taken every 2 h.

### 2.3 Diet formulation

**Table 2** shows the ingredients used in the control and experimental diets formulated in this experiment. Diets were prepared, dried at 60°C, and stored. *Bacillus* sp1, sp2, and sp3 strains were added to feed formulation at 2 × 10^6^ CFU/g.

**Table 2:** Functional feeds formulated for Nile tilapia cultures.

Ingredients	Control group (without probiotics)	Control diet + *Bacillus* sp1	Control diet + *Bacillus* sp2	Control diet + *Bacillus* sp3
Probiotic 2 × 10^6^ CFU/g	0%	1%	1%	1%
Fish meal	10%	10%	10%	10%
Soy meal	40%	40%	40%	40%
Corn meal	16%	16%	16%	16%
Wheat meal	16%	16%	16%	16%
Gelatin	2%	2%	2%	2%
Corn gluten	2%	2%	2%	2%
Vegetable oil	12%	12%	12%	12%
Others	2%	1%	1%	1%
Total	100%	100%	100%	100%

### 2.4 Experimental design

Juveniles (180) of Nile tilapia (33.44 + 0.05 g) were obtained from a local fish farm (Baja California, México) and acclimated for 2 weeks in 2,000-L-water-capacity tanks at 25°C. Fish were randomly distributed in 12 tanks with 200-L water capacity, fed twice a day until satiation with corresponding diet, and parameters were maintained at the following conditions: temperature: 25 ± 2°C, Optical Density (OD): 6.8 ± 0.3 mg/L, pH: 7.4 ± 0.7). After 40 days, growth performance was measured using the next equations:


$$WG=\frac{FWm-IWm}{FIW}*100$$

$$SGR=\frac{LnFW-LnIW}{t}*100$$

TGC=[(√FW− √IW)/(temperature (°C)x t(days))]*100

$$DFI=\sum^{}In\;[total\;feed\;consumed/\#\;of\;fish\;]/t$$

IIW=FW/IW

where FW is the final weight, IW is the initial weight, m is the mean, and t is the time.

### 2.5 Challenging test with *A. hydrophila*

After 40 days of growth, all fishes were inoculated intraperitoneally with 1 ml of *A. hydrophila* solution using 2 × 10^7^ CFU/ml, mortality was recorded, and percentage was calculated using the following equation:


$$\begin{array}{c} Cumulative\;mortality\\ =\left(Total\;number\;of\;dead\;fish\right)\\ ÷\left(Total\;of\;injected\;fish\right)*100 \end{array}$$

### 2.6 Statistical analysis

Data obtained from growth performance were analyzed by one-way analysis of variance (ANOVA) to determine significant variations. Differences between groups were calculated with Tukey’s test. Data were expressed as a mean ± standard error (SE). GraphPad Prism 9 Software (LLC, Boston, MA, USA) was used to perform statistical analyses.

## 3 Results

### 3.1 *A. hydrophila* characterization

#### 3.1.1 Virulence gene detection

**Figure 1:**
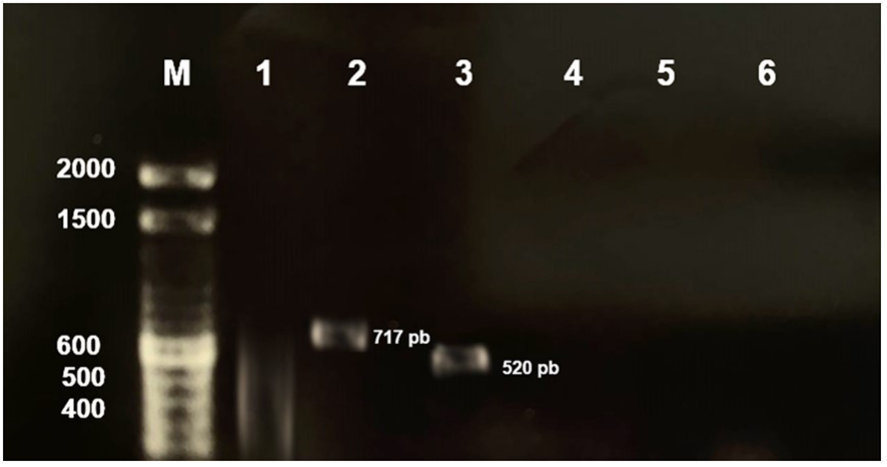
Detection of *A. hydrophila* CAIM675 virulence genes in 1.2% agarose gel. Electrophoresis of PCR products revealed existence of *hylA* (717 pb) and *ahpB* (520 pb) virulence genes. (M) Molecular marker, (1) *act*, (2) *hylA*, (3) *ahpB*, (4) *ast*, (5) *ser*, and (6) *aerA*.

To evaluate the strain capacity of *A. hydrophila* to produce virulence factors, six pairs of oligonucleotides were designed (**Table 1**). PCR results showed that this strain contain *hylA* (717 bp) and *ahpB* (520 bp) virulence genes reported with hemolytic and protease activity, respectively (**Figure 1**).

#### 3.1.2 Growth kinetic and virulence factor production

**Figure 2:**
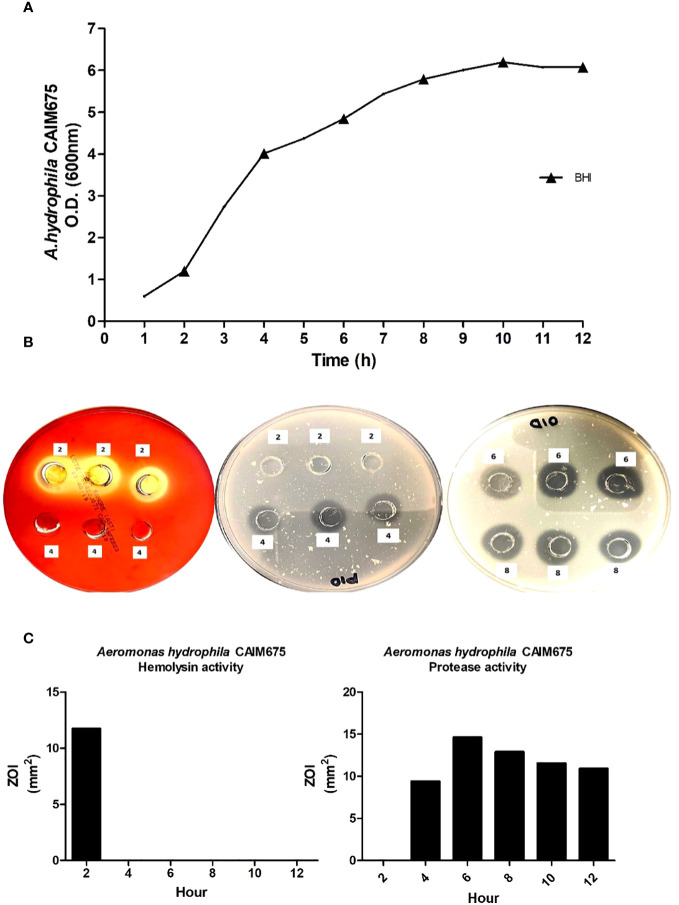
**(A)** Growth kinetic and VF detection (▴) in *A. hydrophila* CAIM675 cultured in BHI medium. The bacterium was cultivated at 37°C and 200 rpm. Growth was followed for 12 h, and samples were collected throughout the exponential growth phase. **(B)** Assessment of hemolytic and proteolytic activities in *A. hydrophila* supernatants; plates were incubated at 30°C for both 24 h, and assays were performed in triplicate. **(C)** Hemolytic and proteolytic activities in *A. hydrophila* CAIM675 EPCs; enzymatic halos were measured in mm^2^, and corresponding values were recorded.

The growth of *A. hydrophila* in BHI medium is shown in **Figure 2A**. To evaluate virulence factor production, samples were taken every 2 h throughout the growth curve. Hemolysin activity was observed only at the early exponential growth phase and does not appear again (**Figure 2B**). Protease activity began just after hemolysin activity ended, and it lasted several hours (**Figure 2B**) showing its highest proteolytic activity at 6 h of culture initiation (**Figure 2C**).

#### 3.1.3 Median lethal dose identification (LD_50_)

**Figure 3:**
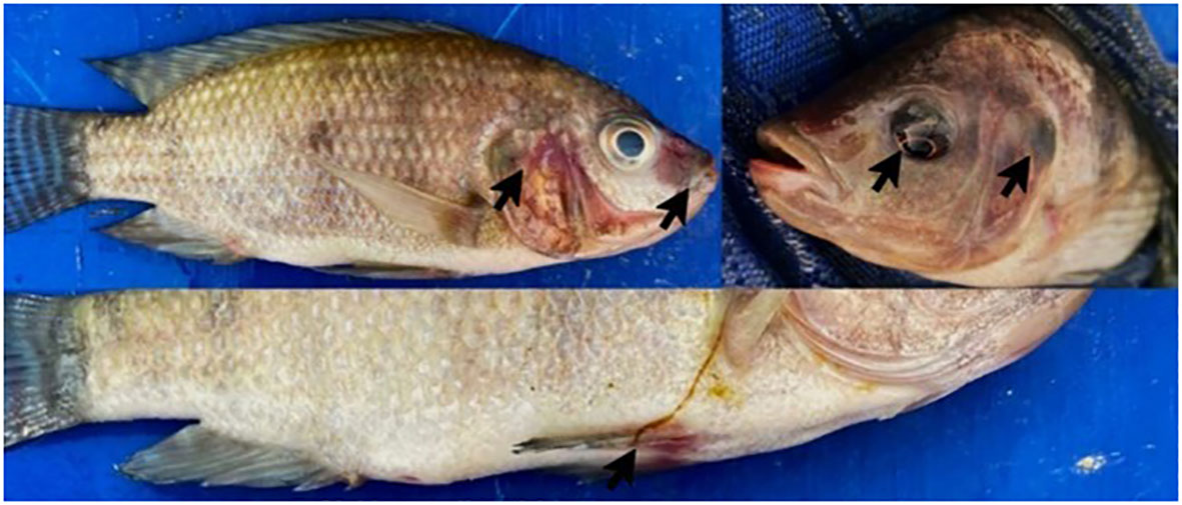
Nile Tilapia infected with *A. hydrophila* CAIM675. Arrows indicate typical symptoms and signs of *A. hydrophila* infection, such as necrosis, hemorrhage, and exophthalmia.

Seven days after the inoculation of pathogen, the fishes started to show typical symptoms (hemorrhages, exophthalmia, necrosis, ulcers) of *A. hydrophila* infection (**Figure 3**). LD_50_ in the control group was reached after 13 days of infection using 2 × 10^7^ CFU/ml of the pathogen bacterium.

### 3.2 *Bacillus* strain characterization

#### 3.2.1 *Bacillus* strain identification

**Figure 4:**
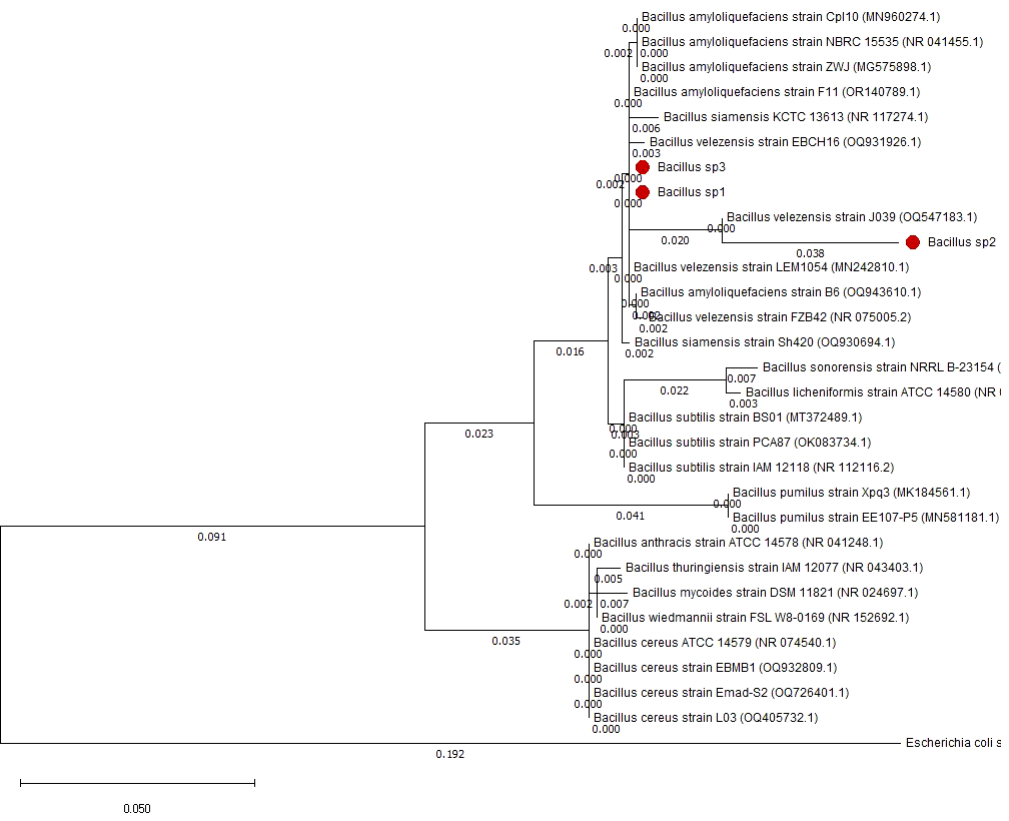
Phylogenetic tree made with 16S rDNA sequences to identify *Bacillus* sp1, sp2, and sp3 strains.

*Bacillus* sp1, sp2, and sp3 strains were selected from a group of 30 sporulating bacteria due to their capacity to grow in soybean products and inhibit *A. hydrophila* in agar plates. After the selection, *Bacillus* strains were grown overnight in LB liquid medium. DNA was extracted, and 16S rDNA genes were amplified. Its sequence was deposited in the PubMed database (sp1 OR504279, sp2 PP229194, and sp3 OR504281). Phylogenetic identification shows that strains sp1, sp2, and sp3 are closely related to *Bacillus velezensis* and *Bacillus amyloliquefaciens*; species that share almost 100% identity belong to the GRAS group of *Bacillus subtilis* (Huynh et al., 2022) (**Figure 4**).

#### 3.2.2 *Bacillus* antimicrobial activity

**Figure 5:**
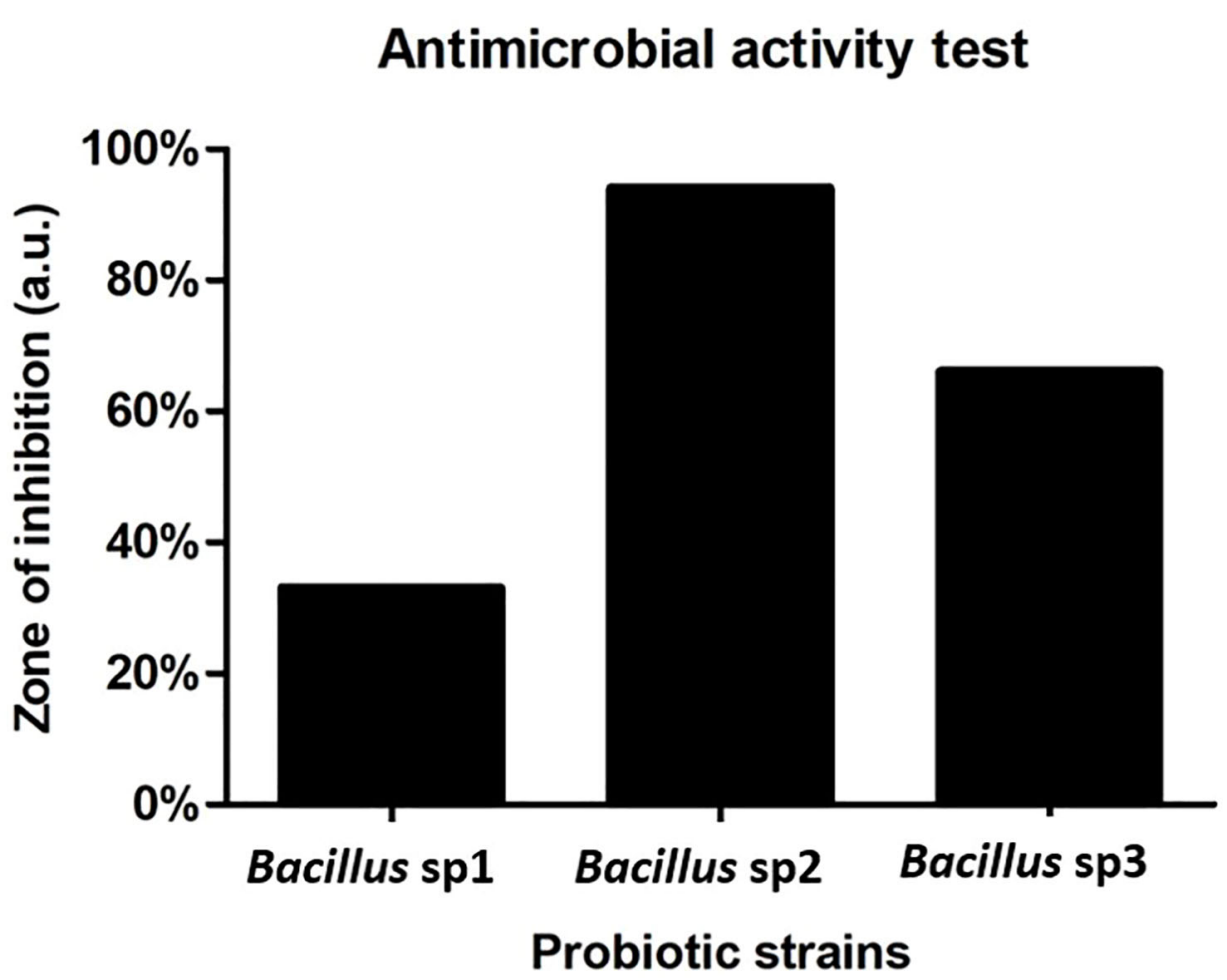
Antimicrobial activity of *Bacillus* strains against *A. hydrophila* CAIM675. *Bacillus* sp2 produced three- and twofold more activity than sp1 and sp3, respectively.

**Figure 5** represents the antimicrobial activity produced by *Bacillus* sp1, sp2, and sp3 strains against *A. hydrophila*. The results show that the sp2 strain produced the highest inhibition activity against the pathogen bacteria producing three- and twofold more activity than the sp1 and sp3 strains, respectively.

#### 3.2.3 *Bacillus* enzymatic activity

**Table 3** shows the growth and enzymatic activity of *Bacillus* sp1, sp2, and sp3 strains in substrates commonly used in aquafeed formulation. The three strains produced similar growth in all the assayed substrates; however, degradation activity produced by these strains was slightly different among them.

**Table 3:** *Bacillus* enzymatic activity assays on the most used substrates in aquafeed formulation.

	*Bacillus* sp1		*Bacillus* sp2		*Bacillus* sp3	
Medium	Growth	Halo	Growth	Halo	Growth	Halo
Wheat	11.2	7.0	11.0	6.2	11.1	5.7
Corn	10.8	8.4	10.9	8.0	10.3	5.0
Soy	11.5	6.8	11.7	6.9	10.5	5.7
Starch	8.6	3.6	8.8	3.2	8.2	3.0
Skim milk	5.6	8.2	5.4	8.3	5.1	8.3
Soy oil	6.6	−	6.8	−	6.3	−

Values are presented as (mm^2^). Values marked as (−) do not present growth.

#### 3.2.4 *Bacillus* antibiotic resistance

**Table 4** shows that only *A. hydrophila* and *Bacillus cereus* that was used as the positive control grew in streptomycin and ampicillin, respectively. However, neither sp1, sp2, nor sp3 grew in any of the assayed antibiotics.

**Table 4:** Antibiotic resistance test following CSLI and EFSA guidelines of *Bacillus* and *A. hydrophila* CAIM675 strains.

Antibiotic/Strain	*A. hydrophila* CAIM675	*Bacillus* sp1	*Bacillus* sp2	*Bacillus* sp3	*Bacillus cereus* *(+)*
Ampicillin	−	−	−	−	+
Chloramphenicol	−	−	−	−	−
Oxytetracycline	−	−	−	−	−
Erythromycin	−	−	−	−	−
Streptomycin	4	−	−	−	−
Clindamycin	−	−	−	−	−
Cephalexin	−	−	−	−	−
Doxycycline	−	−	−	−	−

Values are presented in mm^2^. Values marked as (−) do not present growth; values marked as (+) present growth.

#### 3.2.5 *Bacillus* sp2 strain growth curve

**Figure 6:**
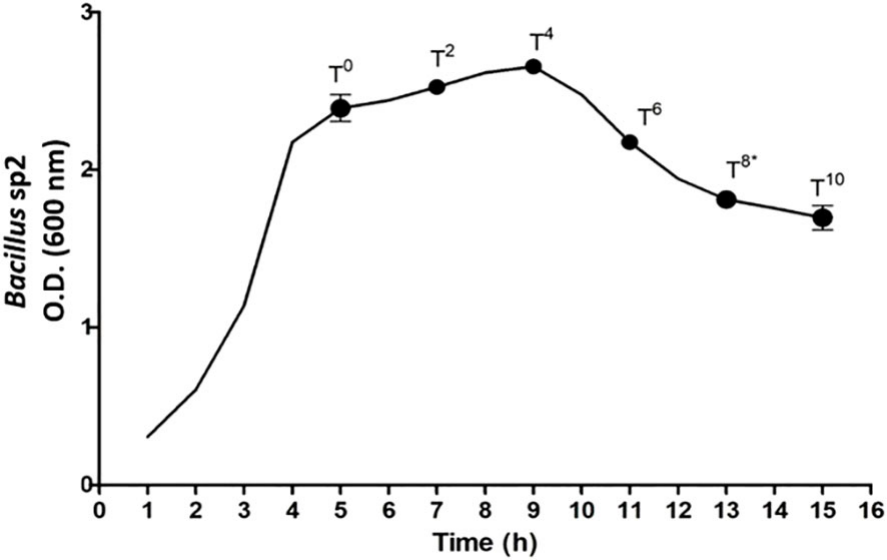
*Bacillus* sp2 strain growth curve in Schaeffer medium. T_0_ indicates the end of exponential growth, and sporulation process began. T_8_ indicates the end of the sporulation process.

**Figure 6** shows the growth curve obtained for sp2 *Bacillus* strain in Schaeffer medium. At T_0_, the exponential growth ended, and the sporulation process began. T_8_ indicates the end of the sporulation process and sampling time for diet preparation. The growth of *Bacillus* sp1 and sp3 was omitted due to a similar behavior.

### 3.3 Nile tilapia growth performance

**Figure 7:**
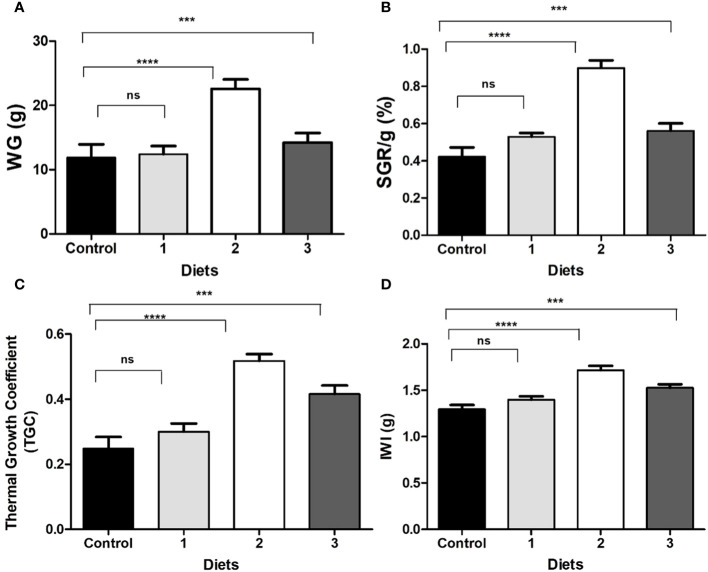
**(A)** Gained weight (WG); **(B)** specific growth rate (SGR); **(C)** thermal growth coefficient (TGC); **(D)** initial weight increment (IWI) of Nile Tilapia fed with control diet and functional diets for 40 days. (Control) Diet without *Bacillus*, (1) *Bacillus* sp1 diet, (2) *Bacillus* sp2 diet, (3) *Bacillus* sp3. Values are means ± SE of triplicate. *Groups are statistically different (*p*< 0.05), and (ns) are not statistically different.

After 40 days of feeding, the fish were measured, and the results were recorded. **Table 5** shows the weight gained, specific growth rate, and other growth parameters that were analyzed in the experiment. **Figure 7** shows that most of these parameters were significantly improved in the *Bacillus* sp2 group with respect to the control, and sp1 and sp3 experimental groups (**Figure 7**).

**Table 5:** Growth performance of Nile Tilapia 40 days after diet supplementation with the *Bacillus* strains.

Parameters	Control	*Bacillus* sp1	*Bacillus* sp2	*Bacillus* sp3
WG (g)	11.861 ± 2.076	12.413 ± 1.262	22.599 ± 1.44^****^	14.209 ± 1.493^*^
WG (%)	30.62	36.79	67.83	55.10
SGR (%)	0.422 ± 0.050	0.530 ± 0.020	0.899 ± 0.041^****^	0.561 ± 0.040^***^
TGC	0.248 ± 0.036	0.300 ± 0.025	0.518 ± 0.021^****^	0.416 ± 0.026^***^
DFI (g)	2.211	1.777	1.9212	1.802
IWI (g)	1.296 ± 0.045	1.398 ± 0.036	1.718 ± 0.046^****^	1.526 ± 0.038^***^

*All values are presented as triplicate means ± SE. Values with (*) are significantly different (p< 0.05). WG, weight gained; SGR, specific growth rate; TGC, thermal growth coefficient; DFI, daily feed intake; IWI, initial weight increment.

### 3.4 Challenging test with *A. hydrophila*

**Figure 8:**
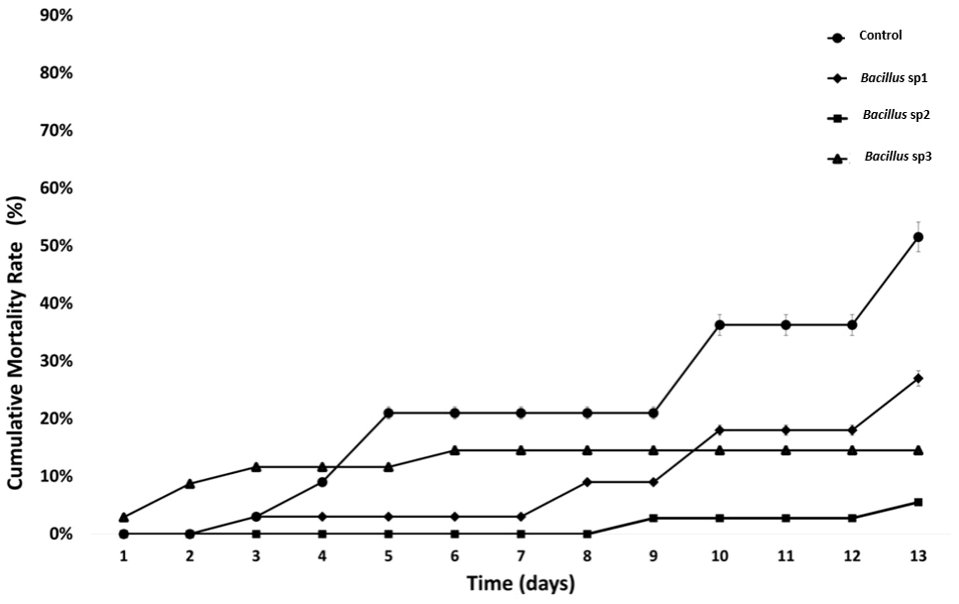
Cumulative mortality rate (%) in Nile Tilapia fed with control and functional diets, and challenged with *A. hydrophila* CAIM675 for 15 Days. The control group reached LD_50_ on the 13th day.

Fishes infected with *Aeromonas hydrophila* after 40 days of feeding were followed for an additional 15 days (**Figure 8**). The results show that the control group reached LD_50_ after 13 days of initial infection. Concurrently, the cumulative mortality of *Bacillus* sp1, sp2, and sp3 reached 25%, 5%, and 15%, respectively, on the same day (**Figure 8**).

## 4 Discussion

Nile Tilapia is susceptible to pathogen bacteria, such as *A. hydrophila*, that induce disease outbreaks and economic losses on intensive culture systems (Kayansamruaj et al., 2014). *A. hydrophila* produce VFs, such as hemolysin, protease, and lipase enzymes, which have been reported as responsible for Nile Tilapia tissue damage and death. Hemolytic and proteolytic activities in tilapia cultures have been directly related to *A. hydrophila* infections (El-Bahar et al., 2019). It is reported that hemolysin is primarily responsible for tissue damage, enteritis, hemorrhages, and anemia; nevertheless, Kanai and Wakabayashi (1984) demonstrated that *A. hydrophila* proteases also produce hemorrhage and muscle necrosis in fish, as does hemolysin. In this sense, the *A. hydrophila* strain used in this work amplified an *hylA* gene, which has been related to hemolysin activity, and an *ahpB* gene, which has been associated with metalloprotease activity (**Figure 1**) (Rasmussen-Ivey et al., 2016). The *hylA* gene product is a cytolytic pore-forming toxin secreted by *Aeromonas* that binds to cell membrane receptors and induces hemolysis (Tomás, 2012; Dubey et al., 2022). On the other hand, the *ahpB* gene product has been related to virulence activity in rainbow trout (Cascón et al., 2000). Both hemolysin and protease enzymes are among the most reported virulence factors in *A. hydrophila* isolates (Li et al., 2011). It is known that VF production depends on the *Aeromonas* strain and culture conditions utilized (Tomás, 2012; Beaz-Hidalgo and Figueras, 2013). Therefore, the culture conditions required to produce virulence factors in *A. hydrophila* CAIM675 strain were investigated in this work (data not shown). BHI medium at 30°C and 200 rpm were identified as the optimum conditions to grow this pathogen (**Figure 2A**). The *A. hydrophila* strain grown under these conditions reached higher optical density compared to those of previous reports (Khalil and Mansour, 1997; Sahu et al., 2011). In addition, VF and their specific production time were characterized. In this sense, hemolysin activity was found only at the beginning of the culture, while protease activity was maintained throughout the exponential growth phase (**Figure 2B**). Some authors have reported that *Aeromonas* can produce alpha-hemolysin during the entire exponential growth phase and beta-hemolysin only at the first hour (Bernheimer and Avigad, 1974; Ljungh et al., 1981; Asao et al., 1986; Tomás, 2012).

In this work, unlike hemolysin production, proteolytic activity was recorded throughout the exponential growth and early stationary phase (**Figure 2B**), as was reported by Khalil and Mansour (1997). In addition, the maximum proteolytic activity was obtained 6 h after culture initiation (**Figure 2C**). Therefore, LD_50_ assay on Nile Tilapia was performed using this culture sample (**Figure 3**).

**Figure 9:**
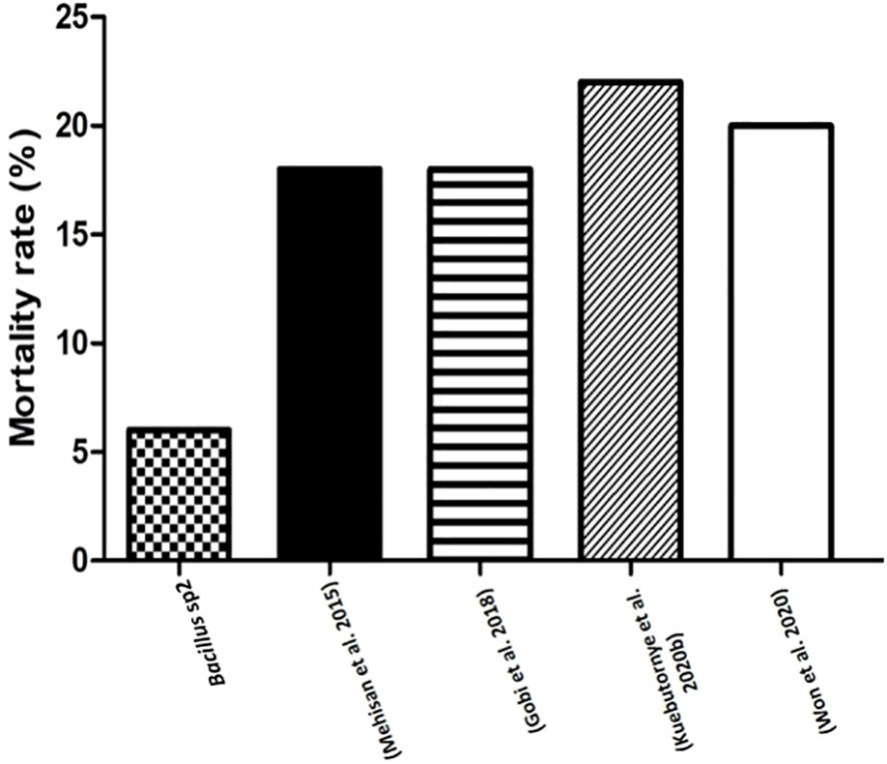
Comparative mortality rates (%) of Nile Tilapia challenged with *A. hydrophila*. This graph compares mortality rates of Nile Tilapia fed with sp2 diet and other reported diets with *Bacillus* strains, and challenged with *A. hydrophila*.

*Bacillus* species have been used to inhibit the virulence factor effects of *A. hydrophila* on Nile Tilapia cultures (Mehisan et al., 2015; Gobi et al., 2018; Kuebutornye et al., 2020b; Won et al., 2020). In this study, the antimicrobial activity of *Bacillus* sp1, sp2, and sp3 strains was phylogenetically identified (**Figure 4**) and evaluated *in vitro* against *A. hydrophila* (**Figure 5**). This figure shows that *Bacillus* sp2 was the most effective in inhibiting *A. hydrophila* development, producing 60% and 30% more antimicrobial activity than sp1 and sp3 strains, respectively. In addition, Nile Tilapia fed with a functional feed supplemented with the *Bacillus* strains and high levels of soybean meal was infected with *A. hydrophila* CAIM675 strain after 40 days of feeding. In this experiment, groups fed with *Bacillus* sp1 (25%), sp2 (5%), or sp3 (15%) strain showed less cumulative mortality compared to the control group (50%) (**Figure 8**). Therefore, *Bacillus* sp2 was the most effective strain in protecting Nile Tilapia from *A. hydrophila* infections. In this sense, *in vivo* results are in agreement with *in vitro* results mentioned above (**Figure 5**). The *Bacillus* capacity to protect Nile Tilapia cultures against *A. hydrophila* has shown good results (Mehisan et al., 2015; Gobi et al., 2018); however, the mortality (5%) of the sp2 group has been the lowest rate reported until now (**Figure 9**).

Antimicrobial peptides, lytic enzymes, organic acids, quorum quenching, nutrient competition, and host immunostimulation, are mechanisms used by *Bacillus* species to control pathogen development . In this sense, some authors have reported the capacity of *Bacillus* peptides to control *A. hydrophila* in Nile Tilapia cultures (Banerjee et al., 2017; Feliatra et al., 2018).

**Figure 10:**
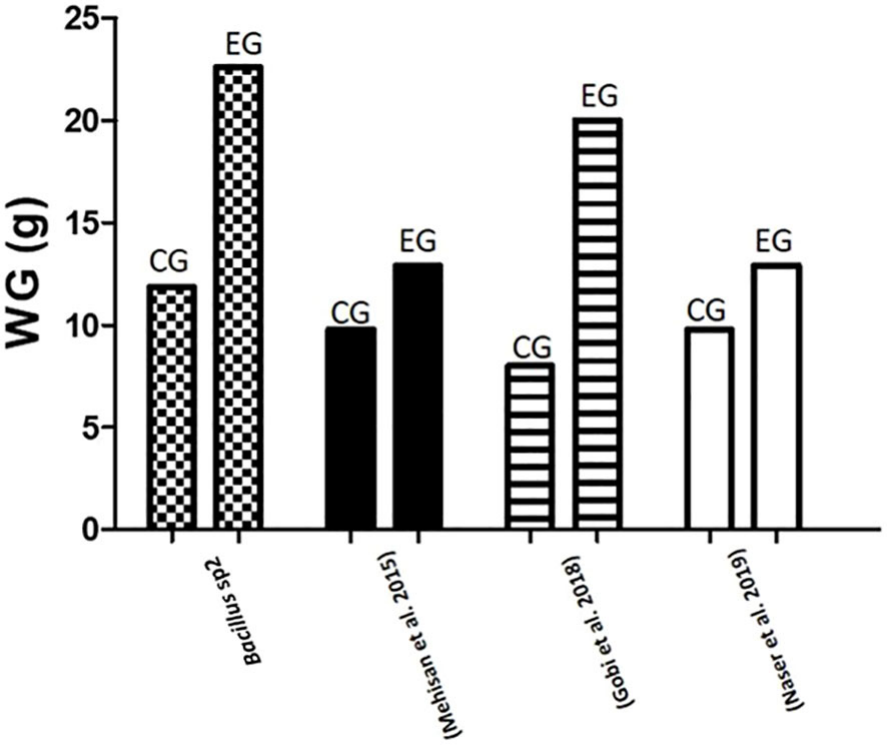
Comparative gained weight (g) in Nile Tilapia experiments. This graph compares results of weight gain in Nile Tilapia cultures fed with control diet (CG) and diet supplemented with sp2 *Bacillus* strain (EG) against other similar experiments reported in the literature.

Moreover, to demonstrate the capacity of *Bacillus* to protect Nile Tilapia from *A. hydrophila* infections, *Bacillus* strains used in this work also showed the capacity to improve growth performance of these fishes (**Table 5**). Thus, diets supplemented with *Bacillus* sp1 (36%), sp2 (67%), and sp3 (55%) strains showed better growth performance than the control group (30%) without *Bacillus* especially the *Bacillus* sp2 group, which improved all parameters (**Table 5** and **Figure 7**). Similar findings have been reported on Nile Tilapia when *Bacillus coagulans* (Mehisan et al., 2015), *Bacillus amyloliquefaciens* (Naser et al., 2019), and *Bacillus licheniformis* (Gobi et al., 2018), were supplemented to Nile Tilapia diets. Nevertheless, the *Bacillus* sp2 group produced better results as it duplicated the weight of the Tilapia in comparison with the control group (**Figure 10**). It is important to mention that even when sp1, sp2, and sp3 induced different growths in Tilapia and inhibited *A. hydrophila* with different capacities, they were isolated from the same fermented soybean sample. In this sense, this could be the reason they are so phylogenetically related (**Figure 4**). Results obtained in this work highlight the properties of the sp2-isolated strain to inhibit *in vitro* development of *A. hydrophila* (**Figure 5**) and reduce pathogen infection capacity in Tilapia (**Figure 9**). In addition, the sp2 strain induced better growth performance than the other reported *Bacillus* strains (**Figure 10**).

FAO (2023) suggested the concentration of macronutrients of Nile Tilapia diet as follows: protein 30%–35%, fat 10%–15%, and carbohydrate 40%–50%. Therefore, diets formulated in this work followed this recommendation (**Table 2**). The *Bacillus* species can grow in a great diversity of nutrient sources due to its capacity to produce protease, carbohydrase, and lipase enzymes (Olmos et al., 2020). In this experiment, the *Bacillus* strains grew well in vegetable sources such as soybean meal (**Table 3**). Soybean products have been used as fish product replacement to prevent environmental contamination, overfishing, and high prices (Tacon and Metian, 2008; Olmos Soto, 2017). However, soy and other leguminous plants contain antinutritional factors, such as trypsin inhibitors, lectins, and toxic oligosaccharides, with capacity to produce tissue damage and animal death. In this sense, some *Bacillus* species have been reported with the capacity to break down antinutritional factors contained in soy products (Shi et al., 2017; Giliyaru et al., 2018).

**Figure 11:**
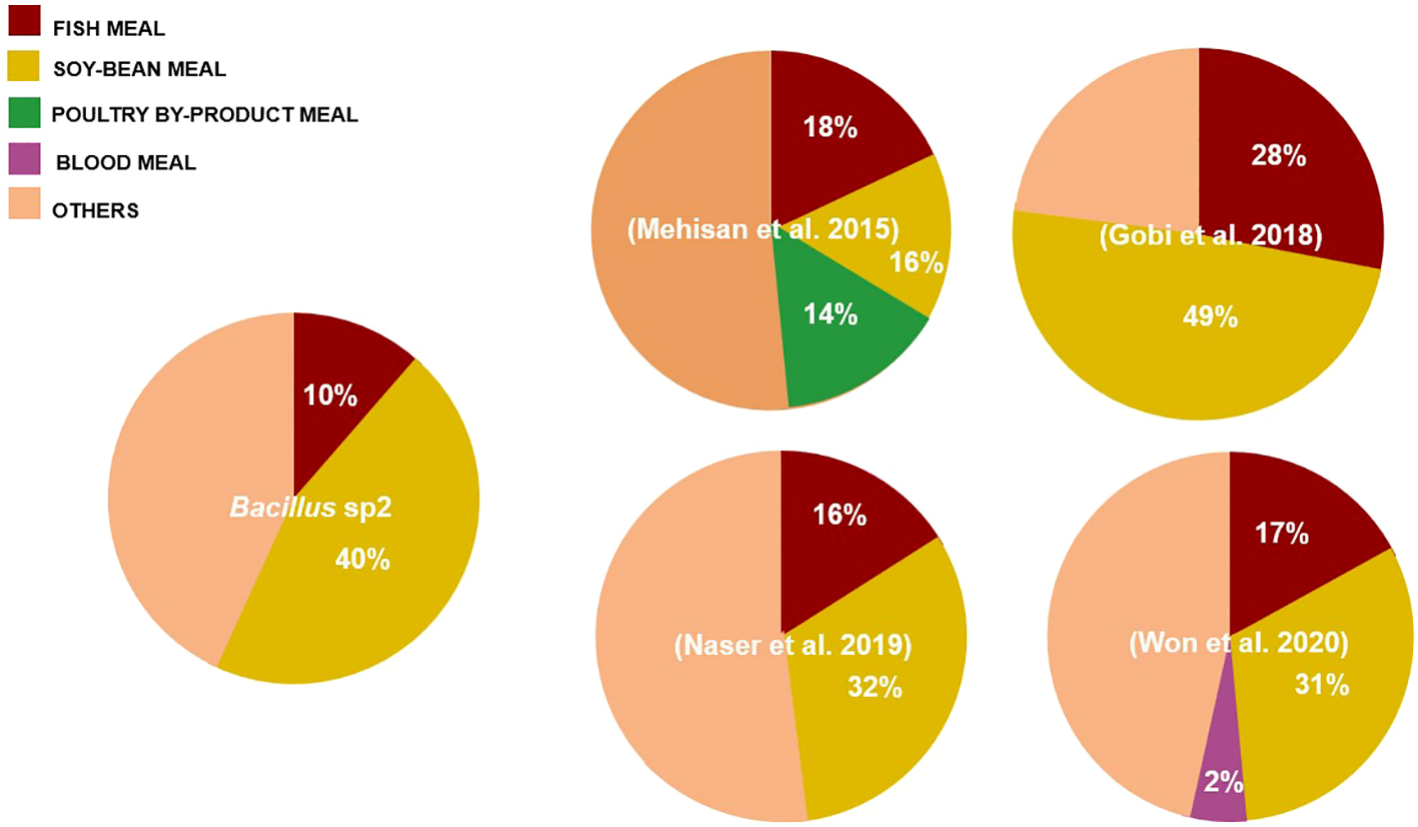
Comparison of diets formulated with *Bacillus* and soybean meal (%) used in Nile Tilapia cultures. The pie chart compares our formulation with respect to formulations used in other experiments.

Some authors have reported aquafeed formulation using soy products and *Bacillus* strains; *Oreochromis niloticus* (Addo et al., 2017), *Pagrus major* (Zaineldin et al., 2021), and *Totoaba macdonaldi* (Olmos et al., 2022) are some successful examples. In this sense, the growth performance of Nile Tilapia obtained in this work suggests that *Bacillus* strains, especially *Bacillus* sp2, could alleviate antinutritional factor effects produced by soybean products (**Table 5** and **Figure 7**). Gobi et al. (2018) found a similar growth rate using even more soybean meal; however, the authors used almost three times more fishmeal than was used in this experiment (**Figure 11**).

## 5 Conclusion

*A. hydrophila* produce virulence factors implicated in Nile Tilapia disease outbreaks. Hemolysin and protease enzymes have been identified as responsible for Nile Tilapia tissue damage and death. In this work, *hylA* and *ahpB* gene products seem to be the virulence factors responsible for inducing tissue damage and death in cultured fishes. In the present study, a functional feed was formulated with soybean meal and the *Bacillus* sp2 strain capable of increasing the health status of Nile Tilapia by inhibiting *A. hydrophila* strain development and VF production. In addition, this feed enhanced growth performance in Nile Tilapia as the *Bacillus* sp2 strain had the capacity to grow in a great variety of plant-based substrates such as soybean meal and other leguminous plants. Additionally, the sp2 strain was identified as part of the *B. subtilis* group; therefore, it can be considered GRAS for further commercial feed development.

## Data availability statement

The raw data supporting the conclusions of this article will be made available by the authors, without undue reservation.

## Ethics statement

The animal study was approved by Comité de Ética CICESE, Centro de Investigación Científica y de Educación Superior de Ensenada, Baja California. The study was conducted in accordance with the local legislation and institutional requirements.

## Author contributions

LM: Data curation, Investigation, Methodology, Writing - original draft. VM: Investigation, Methodology, Writing - review & editing. JO: Investigation, Methodology, Supervision, Writing - review & editing.
